# Mitochondrial disease registries worldwide: A scoping review

**DOI:** 10.1371/journal.pone.0276883

**Published:** 2022-10-27

**Authors:** Ammanie Abdul-Fatah, Leila Esmaeilisaraji, Crisel Mae Juan, Martin Holcik

**Affiliations:** Department of Health Sciences, Carleton University, Ottawa, Ontario, Canada; Auckland University of Technology, NEW ZEALAND

## Abstract

**Background:**

Mitochondrial diseases are a large group of genetically heterogeneous and clinically diverse disorders. Diagnosis often takes many years for which treatment may not exist. Registries are often used to conduct research, establish natural disease progression, engage the patient community, and develop best disease management practices. In Canada, there are limited centralized registries for mitochondrial disease patients, presenting a challenge for patients and professionals.

**Objective:**

To support the creation of such a registry, a systematic scoping review was conducted to map the landscape of mitochondrial disease patient registries worldwide, with a focus on registry design and challenges. Furthermore, it addresses a knowledge gap by providing a narrative synthesis of published literature that describes these registries.

**Methods:**

Arksey and O’Malley’s methodological framework was followed to systematically search English-language literature in PubMed and CINAHL describing the designs of mitochondrial disease patient registries, supplemented by a grey literature search. Data were extracted in Microsoft Excel. Stakeholder consultations were also performed with patient caregivers, advocates, and researchers to provide perspectives beyond those found in the literature. These data were thematically analyzed and were reported in accordance with the PRISMA-ScR reporting guidelines.

**Results:**

A total of 17 articles were identified describing 13 unique registries located in North America, Europe, Australia, and West Asia. These papers described the registries’ designs, their strengths, and weaknesses, as well as their tangible outcomes such as facilitating recruitment for research and supporting epidemiological studies.

**Conclusion:**

Based on our findings in this review, recommendations were formulated. These include establishing registry objectives, respecting patients and their roles in the registry, adopting international data standards, data evaluations, and considerations to privacy legislation, among others. These recommendations could be used to support designing a future Canadian mitochondrial disease patient registry, and to further research directly engaging these registries worldwide.

## Introduction

Mitochondrial diseases (MDs) are a large group of clinically and genetically heterogeneous disorders caused by dysfunctional mitochondria [1, 2]. The mitochondria, colloquially known as the powerhouse of the cell, are key cellular organelles that generate energy (in the form of adenosine triphosphate, ATP) through oxidative phosphorylation to support all biochemical reactions of the cell. Prevalence estimates are variable but conservatively, mitochondrial diseases affect at least 1 in 4,300 persons in Europe [3]. MDs can be further divided into two categories, primary and secondary. Primary Mitochondrial Diseases (PMDs) occur when disease causing mutations arise in the mitochondrial or nuclear DNA (mtDNA and nDNA, respectively) and typically impact the proteins involved in oxidative phosphorylation [2, 4]. Currently, there are over 1,000 identified mutations in mtDNA and nDNA with different modes of inheritance (X-linked, autosomal recessive, or maternal) dependent on where and how the mutation came to be [5]. Some examples of PMDs include Friedreich’s Ataxia (FRDA), Leber Hereditary Optic Neuropathy (LHON), Mitochondrial Encephalopathy, Lactic Acidosis, and Stroke-like episodes (MELAS syndrome), and Myoclonic Epilepsy with Ragged Red Fibers (MERRF). On the other hand, Secondary Mitochondrial Diseases result from mutations in genes without an identifiable mitochondrial connection. These gene mutations do not affect the proteins involved in oxidative phosphorylation but exhibit similar features of mitochondrial dysfunctions [6].

Since mitochondrial dysfunctions affect many organs with high energy needs, such as the liver, skeletal and cardiovascular muscles, and the brain, there are many challenges associated with MDs with regards to diagnosis, treatment, and research [2, 7]. Depending on the genetic mutation, MDs can manifest as combinations of hearing loss, seizures, blindness, weakness, neurodevelopmental delays, stroke, and premature death [7]. Due to the diversity of symptoms, patients have reported visiting more than eight physicians on average and that it often takes them years to receive an accurate diagnosis, for which treatment may not be readily available [8]. Furthermore, since many MDs are recognized as rare diseases, patient recruitment and funding of clinical trials can present challenges to research and drug development [9].

To overcome these challenges, patient registries are used as a valuable tool to provide a platform for aggregating data for research purposes and to encourage data sharing among various stakeholders. These registries can generally be defined as databases that collect and house information about patients. The purpose of the registry will typically determine the type of collected data. For example, in addition to capturing general demographic data, a clinical registry would collect information relating to past medical history and health records, while a biorepository would collect and host biospecimens provided by patients [10–12]. Registries can also be used as a means of engaging with the patient community (i.e., patient groups, advocates, organizations) in the collection of Patient Reported Outcomes (PROs, for example quality of life), or collecting contact information with the purpose of recruitment for research studies [10, 11]. A well-established patient registry can facilitate many different types of research, including epidemiological studies, observational studies to gain a clearer understanding of natural disease history and progression, and randomized control trials examining treatment or therapy efficacy [10, 11]. The results of research facilitated by registries can improve patient quality of life by establishing new clinical guidelines based on more efficient diagnostic methods or more effective treatment [13]. On a macro-scale, these different important pieces of evidence inform health policy, such as allowing for program evaluations, health service planning, as well as providing support for public funding of new therapies and treatments [10, 11, 13].

### Canadian context

While it is clear that patient registries are useful and of great importance to the MD community, no formal national or provincial clinical patient registries for MD patients exist in Canada. MitoCanada, a non-profit organization, in partnership with Lumiio, a global digital health company, recently created a Patient Contact Registry. The main goal of the contact registry is to connect MD patients to research opportunities and clinical trials [14]. Similarly, LHON Canada [15], founded in late 2017, is a patient support group that maintains a contact registry for individuals and families. Registered members in the registry can choose to receive information about research initiatives & developments, clinical trials, treatments and/or therapies, volunteer opportunities, fundraising events, assistive technology, and advocacy initiatives. Members can also connect with others through the registry. These platforms can be used to provide contact information and help identify patients with MD, but further work is needed to gain a better understanding of the MD landscape in Canada. Although these individual efforts provide great service to MD patient community, a broader, national-level initiative is needed.

### Objectives

An understanding of what has already been established internationally is essential before trying to build a comprehensive registry in Canada. As a first step to establishing this fully realized MD patient registry, a scoping review was conducted to undertake an environmental scan of the literature surrounding mitochondrial disease registries. This is the first comprehensive review conducted for registries for this group of diseases. The objectives of this work were established through consultation with various stakeholders (patient caregivers, advocates, researchers, MITO2i, and MitoCanada). There are three main objectives for this review. Firstly, to evaluate the extent of the research available on existing mitochondrial disease registries worldwide. Secondly, to identify gaps in the literature pertaining to designing and establishing such registries. Finally, to summarize, make recommendations, and disseminate our findings back to all interested stakeholders within the wider MD community.

## Methodology

A scoping review was conducted using the six-step methodological framework for scoping reviews outlined by Arksey and O’Malley [16] with supplemented recommendations from Levac et al. [17]. These six stages include: identifying the research question; identifying relevant studies; study selection; charting the data; collating, summarizing, and reporting the results; and the optional consulting exercise. Additionally, Preferred Reporting Items for Systematic reviews and Meta-Analyses extension for Scoping Reviews (PRISMA-ScR) reporting guidelines by Tricco and colleagues [18] were consulted to ensure all required items were reported in this review (S1 Table).

### Stage 1: Research questions

After consultation with stakeholders, including patient advocates, and establishing objectives, the following research questions were articulated:

1. What are the existing MD registries worldwide?
2. What kind of information can be found in the existing MD registries?
3. Who are the end users of the current MD registries worldwide?
4. What are the gaps in the literature regarding the design and development of MD registries worldwide?

### Stage 2: Relevant studies identification

To identify the relevant literature, a comprehensive search was conducted in consultation with an information specialist, Carleton University’s Health and Biosciences librarian. Two academic databases were chosen for their relevance to the topic of this scoping review: PubMed and CINAHL. Database-specific search strategies were established collaboratively between the authors and librarian. External partners MitoCanada and MITO2i were consulted to ensure that the breadth of PMDs was captured with the chosen search terms. The final strategy was peer-reviewed by the librarian using the Peer Review of Electronic Search Strategies (PRESS) guideline [19]. The details of the search strategy, including key words and MeSH (Medical Subject Headings) terms, are provided within the supplemental information (S2 Table). Initial searches in each database were conducted from inception until the search date on March 24^th^, 2021 and updated once more on January 26^th^, 2022. In the cases where full-text articles were not readily available, we contacted study authors directly to request a copy.

The systematic database search was supplemented with a grey literature search, using the Google general search engine in February 2021 to identify any additional MD registry related publications.

### Stage 3: Study selection

#### Inclusion and exclusion criteria

Articles of interest included in this review were papers that described PMDs registries (see S3 Table for the list of included PMDs) that were available in English (based on feasibility, as we had limited language translation services). Since Retinitis Pigmentosa (RP) is one of the most common groups of vision disorders with types falling within the PMD umbrella [20], we included any non-MD-specific registries (e.g., eye diseases registries) that included RP patients for their eye presentations. We excluded studies that only mentioned an MD registry for recruitment of subjects or for epidemiological analyses without any explanation on the structure of the registry. We also excluded case reports, case series, animal studies, and descriptive genomic studies. Additionally, we excluded genomic registries (i.e., housing bioinformatic mitochondrial DNA data) or databases of hospital records. These criteria were established *ad hoc* as we became more familiar with the literature.

#### Study screening

We performed two levels of screening against these eligibility criteria using the online systematic review software program Distiller Systematic Review [21]. For title and abstract screening, we used the liberal accelerated method where only one reviewer is required to include citations for further assessment at full-text screening, and two reviewers are needed to exclude a citation [22]. This was to ensure maximal inclusion. The full-text screening that followed was performed in duplicate and independently. Any disagreements among reviewers were resolved through consensus or third-party adjudication. Both levels of screening were preceded by pilot exercises to ensure consistent application of inclusion and exclusion criteria by reviewers. Each round of the pilot exercise for the title and abstract screening included at least 50 articles, while full-text screening pilot rounds included at least 10 articles. Kappa statistic values for inter-rater reliability scores were calculated after each round, and pilot testing continued until the scores reached a minimum of 0.80 (which is considered satisfactory) [23]. We achieved overall Kappa statistic values of 0.92 (95% confidence interval: 0.83, 1.00) for title and abstract screening after three rounds, and 0.89 (95% confidence interval: 0.67, 1.00) for full-text screening after two rounds. These scores were calculated using the Online Kappa Calculator [23].

### Stage 4: Data charting

Data from included studies were charted into a data extraction form that was created in Microsoft Excel (2021) by the reviewers. The validity of the form and the consistency of the reviewers was verified in a pilot exercise using 20% of the included articles. The rest of the articles were data mapped by one reviewer and verified by a second reviewer. Any disagreements were resolved by consensus. The charted information, if available, included: general characteristics of the registry (e.g., name of the registry, country of conduct, source of funding, year of establishment, associated registry website, etc.), patient information (e.g., who makes up the population of the registry, diagnosis, etc.), registry management (e.g., consent obtainment, type of information collected, data security and confidentiality, types of staff involved, etc.) and registry output (e.g., end users, strengths and weaknesses). A full list of all items that were charted can be found in the supplemental information (S4 Table).

### Stage 5: Results collection, summarization, and reporting

Consistent with Arksey and O’Malley’s [16] framework for scoping reviews, we reported an overview of all material reviewed and didn’t make any attempt to synthesize any evidence. Charted information was collated and grouped based on the emerging themes and reported narratively.

### Stage 6: Stakeholder consultation

Stakeholder consultations took place with MD researchers, patient advocates, and caregivers (*N* = 7) in the forms of interviews or general discussions. The purpose of these consultations was to gain an understanding of the perspectives of the MD community, to see if there were common viewpoints that arose from these different stakeholders, and to compare their experiences and expectations of what an ideal registry should entail with what is currently established across the world. All stakeholders provided written informed consent. Furthermore, all stakeholders had the opportunity to review meeting minutes and provide feedback or edits. Meeting minutes were then thematically analyzed using an inductive semantic approach [24]. All identifying information was removed and identified themes were reported.

## Results

**Fig 1:**
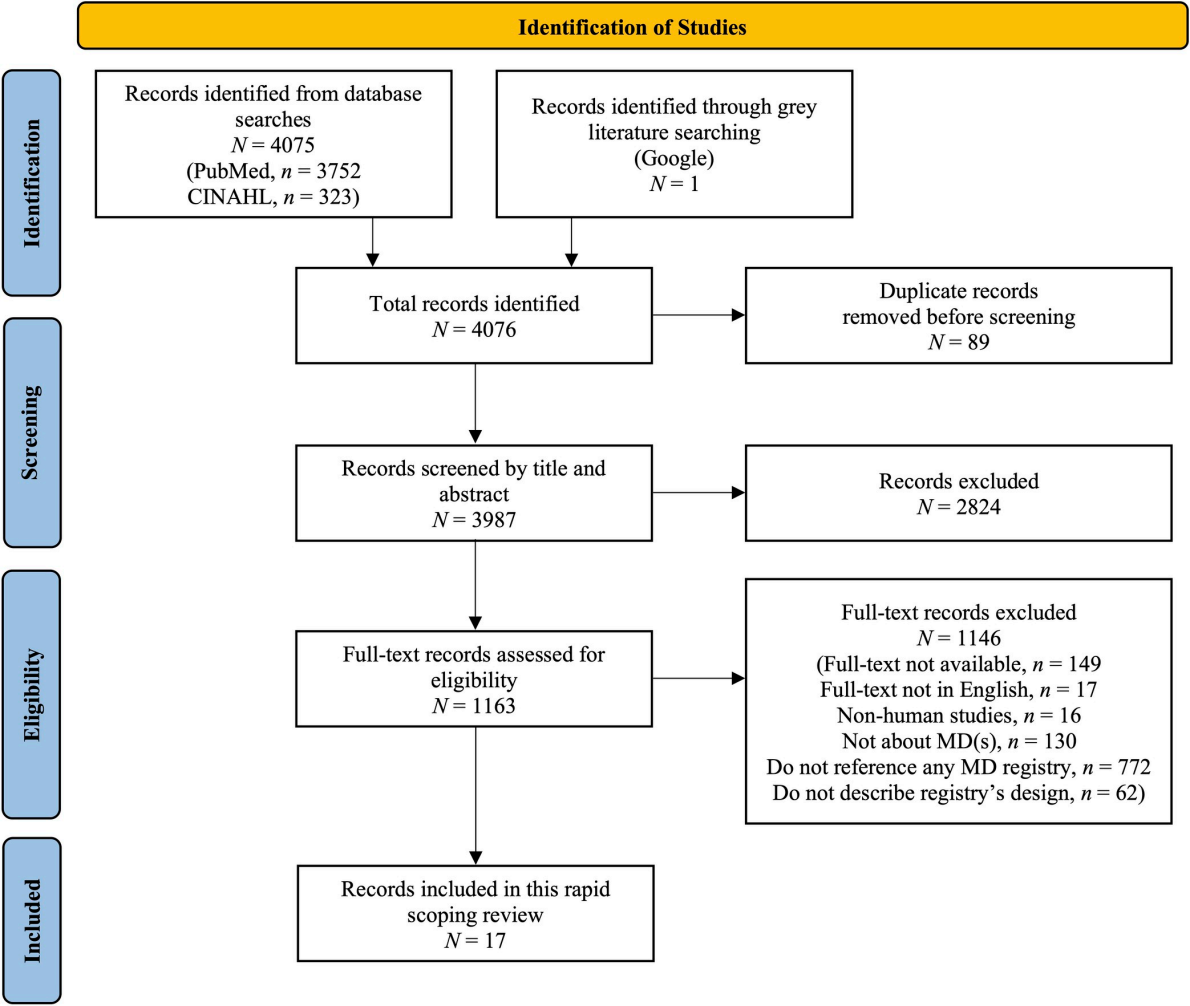
PRISMA flow diagram for study inclusion. This diagram was adapted from Page et al. [25]. MD: Mitochondrial disease.

An initial search was conducted in the PubMed and CINAHL databases in March 2021 that identified 3899 studies. The rerun of the search in January 2022 resulted in 176 new hits. Grey literature searching identified one potentially relevant record for a total of 4076 studies. Duplicate removal reduced this number to 3987 records. Excluding 2824 studies at the title and abstract level led to 1163 studies moving to full-text screening. Reasons for exclusion at this second level of screening included that the full-text was not available in English (*n* = 17), the study population was not human (*n* = 16), the article was not about MDs (*n* = 130), there was no reference to any MD registry (*n* = 772), or we did not have access to full-text of the article (*n* = 149). The remaining 62 studies that were excluded and did not move on to the data charting stage were studies that only mentioned an MD registry but did not provide any details on its structure. As a result, 17 studies passed full-text screening, and moved on to the data charting phase of this scoping review (Fig 1).

Of these 17 included studies, the vast majority were descriptive papers that provided details on registry design as well as the registries’ participant cohorts (*n = 11*) [7, 26–35], while some were epidemiological studies that also provided information pertaining to registry design (*n = 4*) [36–39]. Two studies solely described the registries’ designs without providing details on their respective participant cohorts [40, 41]. All of the included papers reported either their source of funding or that the authors had no conflicts of interest, with the exception of three articles for which it was not clearly reported [29–31].

### General registry characteristics

The 17 included studies in this scoping review described 13 unique MD registries worldwide (Table 1). Four of the registries, the North American Mitochondrial Disease Consortium (NAMDC) Registry [7, 26], the My Retina Tracker Registry [29, 30, 40], the Mitochondrial Disease Community Registry (MDCR) [35], and the Champ Foundation Registry (CFR) [33], were founded in the United States. Two were founded in Australia, which included the Australian Inherited Retinal Disease Register and DNA Bank (AIRDR) [28], and the Western Australian Retinitis Pigmentosa Register [27]. Two more were founded in the Netherlands, including the Netherlands Leber’s Disease Archive [41], and the Department of Ophthalmogenetics of the Netherlands Ophthalmic Research Institute Central Registration [39]. Other registries were founded in Norway (the Norwegian Inherited Retinal Disease Registry) [38], Iran (the Iranian Registry for Inherited Retinal Diseases) [34], Denmark (Danish Retinitis Pigmentosa Register) [36, 37], and the United Kingdom (Bradford’s Low Vision Register) [31]. Finally, the European Friedreich’s Ataxia Consortium for Translational Studies (EFACTS) was a registry that was established by the European Union and the United Kingdom [32]. Furthermore, these identified registries have different levels of geographic coverage. Four of the registries (the NAMDC Registry, the MDCR, the My Retina Tracker Registry, the EFACTS) had international coverage, two had regional coverage (Bradford’s Low Vision Register, Western Australian Retinitis Pigmentosa Register) and the rest had national coverage (Table 1). The NAMDC Registry, the MDCR, and the My Retina Tracker Registry have registered some Canadian patients; the MDCR clearly specified that 27 of their patients, comprising 2.89% of all registrants, were Canadian MD patients and the My Retina Tracker Registry reported that 14% of their total registrants were Canadian [7, 30, 35]. Although the status of two registries could not be determined [34, 41], the remaining enumerated registries are still active, with the exception of the MDCR [35]. The oldest MD registry identified in this scoping review is the Netherland’s Leber’s Disease Archive [41], while the newest is the CFR, established in 2020 [33].

**Table 1:** Mitochondrial Disease (MD) patient registries’ characteristics.

Registry	MD(s) Covered	Registry Coverage	Year of Establishment	Source of Funding	Associated Organization	Type of Registry	Associated Biorepository (Yes/No)	Purpose	Registry Active (Yes/No)	Registry Participants	Recruitment Source
Australian Inherited Retinal Disease Register and DNA Bank (AIRDR) [28]	IRDs including RP	National	Regionally established in 1984, national expansion in 2009	Government organizations, charity foundations	The DMTP at the Sir Charles Gairdner Hospital, Western Australia	Clinical and geneticregistry	Yes	To provide pooled data to facilitate research, to translate research findings into clinical applications	Yes	Patients and relatives of probands	Hospitals and ophthalmology clinics
Bradford’s Low Vision Register [31]	Vision disorders including RP	Regional	NR	NR	Morley Street Resource Centre	Municipal resident database	No	To support people who have a visual impairment	Yes	People with visual impairment(s) living in Bradford, United Kingdom	The Morley Street Resource Centre
Champ Foundation Registry (CFR) [33]	SLSMDS	NR	2020	Non-profit organization	The Champ Foundation	Patient-mediated clinical registry	No	To improve the understanding of SLSMDS through patient data, and links data from PRO surveys, electronic medical records, biospecimens, and physician reported outcomes	Yes	Patients	Social media and the Champ Foundation
Danish Retinitis Pigmentosa Register [36, 37]	RP	National	1986–1989	Non-profit organization	The Danish Association of the Blind, Data Surveillance of Authority, Ministry of Social Affairs	Clinical registry	No	To establish a database for future research, to determine national prevalence of RP	Yes, at the time of publication	Patients and their families	The National Eye Clinic for the Visually Impaired and the National Institute for the Blind and Partially Sighted, hospitals and private ophthalmology clinics, the National Diagnostic Index for In-patients, and the Danish Association of the Blind
Department of Ophthalmogenetics of the Netherlands Ophthalmic Research Institute Central Registration [39]	Genetic eye diseases including RP	National	1972	NR	Netherlands Ophthalmic Research Institute	Clinicalregistry	No	NR	Yes, at the time of publication	Patients and their families	Referral from clinics and members of Societies for the Blind in the Netherlands
European Friedreich’s Ataxia Consortium for Translational Studies (EFACTS) [32]	FRDA	International (11 European centers within the EFACTS framework)	2010	Government organization	NR	Clinical registry	NR	NR	Yes	Patient	NR
Iranian Registry for Inherited Retinal Diseases [34]	IRDs including RP	National	2016	Government organization, academic institution	Iranian Ministry of Health and Medical Education and Shahid Beheshti University of Medical Sciences	Clinicalregistry	Yes	To determine the prevalence of IRD, to identify the genetic landscape, to provide pooled data to facilitate research collaboration	NR	Patients	Public announcement, patient support groups
Mitochondrial Disease Community Registry (MDCR) [35]	All MDs	International	2014	Non-profit organization	UMDF	Patient experiences and perspectives registry	No	To identify and characterize MDs from the patient perspective to enhance clinical care	No, data collection ended September 2018	Patients and caregivers	Advertising on the UMDF website, using the UMDF email list, and social media
My Retina Tracker Registry [29, 30, 40]	IRD including RP	International	2014	Charity foundations, research organization	Foundation Fighting Blindness	Clinical and genetic registry	No	“To provide anintegrated source of information about, and connection to, all peoplewith an IRD; and to share those data …with researchers and partners” [30 p. 840]	Yes	Patients and their unaffected, genetic relatives	Free-online participation
Netherlands Leber’s Disease Archive [41]	Leber’s Disease	National	Before 1963	Not reported	Netherlands General Association for the Prevention of Blindness	Clinical and pedigree registry	No	To develop archives “containing all the data about the Leber patients and their families living in the Netherlands” [41 p. 1]and facilitate the research	NR	Patient and their families (pedigree)	Clinics and a birth registry
North American Mitochondrial Disease Consortium (NAMDC) Registry [7, 26]	All MDs	International (US, Canada)	2011	Government agencies and organizations, patient’s advocacy groups, non-profit organization.	RDCRN, UMDF, Eunice Kennedy Shriver NICHD, the ODS, and the NCATS.	Clinical registry	Yes	To support and conduct translational research on MDs.	Yes	Patients, including asymptomatic carriers	17 North American medical centers
Norwegian Inherited Retinal Disease Registry [38]	IRDs including RP	National	2016 (although data collection started in 2003)	Not reported	Not reported	Clinical and genetic registry	Yes	To estimate the prevalence of IRD, to support “future clinical trials, treatment selection, and counselling of families” [38 p. 286]	Yes	Patients	Medical records
Western Australian Retinitis Pigmentosa Register [27]	RP	Regional	1984, then expanded to the AIRDR in 2009	Non-profit organization	Western Australian RP Foundation	Clinicalregistry	No	To establish regional prevalence of RP, collect clinical data and natural history data, assess efficacy of RP treatments	Yes, at the time of publication	Patients and their families	Ophthalmologist referral and the Western Australian RP Foundation

DMTP, Department of Medical Technology and Physics; FRDA, Freidrich’s Ataxia; IRD, Inherited Retinal Disease; NCATS, National Center for Advancing Translational Science; NR, Not Reported; ODS, Office of Dietary Supplements; PRO, Patient Reported Outcome; RDCRN, Rare Disease Clinical Research Network; RP, Retinitis Pigmentosa; SLSMDS, Single Large-Scale mtDNA Deletion Syndromes; UMDF, United Mitochondrial Disease Foundation.

Generally, most of the registries were developed to provide an aggregated data source for facilitating research for knowledge generation, and to improve clinical practices for enhancing the quality of care and quality of life of MD patients (Table 1). In addition, four of the registries sought to determine the prevalence of the disease(s) of interest in a specific geographical area and were used for epidemiological studies [27, 34, 36–38].

The source of recruitment varied from referral from hospitals or specialist clinics to online voluntary participation informed by public announcement and media campaigns (Table 1). In terms of disease coverage, the NAMDC Registry [7] and the MDCR [35] were the only two registries where all MDs were included without any restriction as to the type of MD. Five registries focused on a specific type of MD (i.e., RP, FRDA, Leber’s Disease, and Single Large-Scale mtDNA Deletion Syndromes, SLSMDS) [27, 32, 33, 36, 37, 41]. Lastly, six of the registries are for general eye diseases that included RP [28, 30, 31, 34, 38, 39], a type of mitochondrial retinopathy [42]. All the registries found in this review, except for the MDCR, were collecting clinical data and most of them were also storing genetic, family history/pedigree, and biochemical data. Some registries, including the NAMDC registry, the Norwegian Inherited Retinal Disease Registry, the Iranian Registry for Inherited Retinal Diseases, and the AIRDR, have a biorepository in addition to the main clinical registry [26, 28, 34, 38]. Other types of registries were also identified to incorporate retrospective data (the Danish Retinitis Pigmentosa Register), natural history data (the NAMDC Registry and the My Retina Tracker Registry) and patient perspectives (the MDCR, the CFR, the My Retina Tracker Registry) [7, 29, 30, 33, 35–37]. A very unique feature of the MDCR [35] was the priority placed on collecting data from patients or caregivers about the impact of the disease on their quality of life, availability of treatment and care and the importance of clinician-patient communication.

The literature included in this review provided information on the sources of funding for nine registries (Table 1) [7, 26–30, 32–37, 40]. While most of these registries were supported by more than one funding source (e.g., charitable foundations, patient advocacy groups, government organizations, and research institutes), the CFR, the MDCR, and the Western Australian Retinitis Pigmentosa Register relied only on a single source of funding [27, 33, 35]. None of the registries reported sponsorship from private for-profit companies. Based on the literature included in this review, the source of funding of the Netherlands Leber’s disease Archive, the Department of Ophthalmogenetics of the Netherlands Ophthalmic Research Institute Central Registration, the Norwegian Inherited Retinal Disease Registry, and Bradford’s Low Vision Register could not be determined [31, 38, 39, 41]. Furthermore, other than the EFACTS and the Norwegian Inherited Retinal Disease Registry [32, 38], the rest of the registries reported being associated with another organization or foundation. For instance, the My Retina Tracker Registry was associated with the Foundation Fighting Blindness and the CFR was working with the Champ Foundation [30, 33, 40]. However, the extent and quality of this cooperation was not described.

### Clinical aspects

A review of the clinical aspects of the registries was done to gain an understanding of what clinical patient information is being collected within the registries and to see how that data differs globally. For recruitment, medical diagnosis of MDs was often used to enroll participants, but the criteria varied depending on the type of MD. In the NAMDC Registry, for example, benchmarks are used for definite, suspected, or unlikely diagnoses according to the NAMDC Research Diagnostic Criteria [7]. Other registries which categorize clinical examination based on certainty include the Danish Retinitis Pigmentosa Register (e.g., certain, possible, and probable) [37] and the Western Australian Retinitis Pigmentosa Register (e.g., affected-definite, affected-probable, affected-possible, and unaffected) [27]. Some registries also included genetic testing as part of their diagnostic process like the Norwegian Inherited Retinal Disease Registry, Iranian Registry for Inherited Retinal Diseases, and the EFACTS [32, 34, 38]. Participants in the AIRDR must have a provisional or confirmed diagnosis of an Inherited Retinal Disease (IRD), with each diagnosis being assigned a confidence level depending on its perceived level of reliability [28].

Most registries collected data pertaining to patients’ demographics, MD diagnosis information, general health, family history and clinical records. The registries that had biorepositories collected biospecimens and recorded additional information like biochemical, molecular, genetic, and pedigree data [7, 26, 28, 32, 34, 38]. In addition, the MDCR, the CFR and the My Retina Tracker Registry captured participants’ subjective experience living with the disease and its impact on their quality of life [30, 33, 35].

### Technical aspects

Various software platforms, terminology standards, and common data elements were used by different registries (Table 2). Data mining tools were included in the NAMDC Registry to facilitate sharing of de-identified data among NAMDC investigators as well as a Master Service Agreement that permits the transfer of biosamples between NAMDC sites and to external entities with the appropriate authorization [7]. Each registry has their own unique diagnostic criteria. For example, the NAMDC Registry developed their own diagnostic criteria in collaboration with different experts in the medical field (e.g., neurology, pediatrics, pathology) while others included genetic testing as part of their process like the Norwegian Inherited Retinal Disease Registry, Iranian Registry for Inherited Retinal Diseases, and the EFACTS [7, 32, 34, 38]. In terms of terminology standards, different types were used within the included registries, such as: the International Classification of Diseases (ICD) 8, 9, 10, and 11; the Systemized Nomenclature of Medicine-Clinical Terms (SNOMED-CT); and the Unified Medical Language System (UMLS), among others (Table 2). Only the My Retina Tracker Registry reported complying with NIH-supported Common Data Elements, which improve the accuracy of database searches and sharing of de-identified data across databases [29].

**Table 2:** Software platforms and standardized measures used by Mitochondrial Disease (MD) patient registries.

Registry	Software Platform	Standardized Measure(s)
Australian Inherited Retinal Disease Register and DNA Bank (AIRDR) [28]	Cyrillic v2.02 (Cyrillic Software, Oxfordshire, UK)	NR
Bradford’s Low Vision Register [31]	NR	NR
Champ Foundation Registry (CFR) [33]	NR	Non-standardized PRO measures, however their surveys were validated with patient community prior to launch
Danish Retinitis Pigmentosa Register [36, 37]	Only indicated that the registry was computer-based	“Official Danish [World Health Organization] coding system, ICD-8 with an extension worked out by the Danish Ophthalmological Society” [37 p. 166]
Department of Ophthalmogenetics of the Netherlands Ophthalmic Research Institute Central Registration [39]	Only indicated that the registry was computer-based	ICD-9, “extended with the British Paediatric Association Classification of Diseases (1979) and the Classification of Disorders of the Eye by Colenbrander (1975)” [39 p. 228]
European Friedreich’s Ataxia Consortium for Translational Studies (EFACTS) [32]	NR	ICD-10, Inventory of Non-Ataxia Signs (INAS), Scale for the Assessment and Rating of Ataxia (SARA), and Spinocerebellar Ataxia Functional Index (SCAFI)
Iranian Registry for Inherited Retinal Diseases [34]	PostgreSQL based on the District Health Information Software (DHIS2), programmed in Java.	Unified Medical Language System (UMLS), ICD-11, Online Mendelian Inheritance in Man (OMIM), Orphanet Rare Disease Ontology (ORDO), and Systemized Nomenclature of Medicine-Clinical Terms (SNOMED-CT).
Mitochondrial Disease Community Registry (MDCR) [35]	“Platform for Engaging Everyone Responsibly (PEER) owned and operated by Genetic Alliance” [35 p. e3]	NR
My Retina Tracker Registry [29, 30, 40]	PatientCrossroads and uses the Patient-Reported Outcomes Measurement Information System®	Global Unique Identifier (GUID) and “NIH-supported Common Data Elements (CDE) for standardized terminology” (Fisher et al., 2016, p. 33)
Netherlands Leber’s Disease Archive [41]	Hollerith system	NR
North American Mitochondrial Disease Consortium (NAMDC) Registry [7, 26]	Only indicated that the registry used “a secure web-based data entry system” [26 p. 2]	Generally not reported, other than the NAMDC Research Diagnostic Criteria that they established
Norwegian Inherited Retinal Disease Registry [38]	MedInsight	ICD-10, ClinVar classification status was used to interpret variants identified from high-throughput sequencing, and population frequencies were used from the ExAC and the gnomAD website
Western Australian Retinitis Pigmentosa Register [27]	Unclear, but indicated that data was stored on a computer in a hospital, written in FORTRAN IV	NR

ExAC, Exome Aggregation Consortium; gnomAD, Genome Aggregation Database; ICD, International Classification of Diseases; NIH, National Institutes of Health; NR, Not Reported; PRO, Patient Reported Outcome; RDCRN, Rare Diseases Clinical Research Network.

Clinical data were collected using a variety of methods, but most commonly through clinical visits, medical records, and surveys. Biosamples were directly collected from individuals by registries with biorepositories (e.g., the AIRDR) once informed consent was obtained [28]. Registries with member clinical sites or resource centers, such as the NAMDC Registry, gather patient information through these sites in person or via web-based remote enrollment [7]. Similarly, the data for the Bradford Low Vision Register have been obtained from the Morley Street Resource Center, which maintains a list of all blind and partially sighted people residing in the Bradford Metropolitan District [31]. In most cases, data were collected by clinicians or researchers through online platforms or paper-based questionnaires, except for patient-driven registries (the MDCR, the CFR, and the My Retina Tracker) in which patients or their caregivers enter their own information and keep track of their own records [29, 33, 35].

### Staff requirements

A few of the included registries indicated the staff that they required to operate. For example, to successfully implement the registry, the NAMDC Registry established an administrative core that provides crucial organizational and strategic support. It includes “an Overall Program Director/Principal Investigator, a Statistical Principal Investigator, a Clinical Team Liaison, a Bioinformatician, and an Administrative Coordinator, with ultimate responsibility for the Program’s scientific, clinical research, and training/educational operations" [7]. As well, a national steering committee was formed by the Iranian Registry for Inherited Retinal Diseases which included eight sub-committees focused on "health terminology and coding, financial and administrative, data entry, quality control and evaluation, statistical analysis and epidemiology, information technology, scientific and research, documentation and website [updates]" [34]. In the case of the My Retina Tracker, the registry was designed alongside 19 advisors from the United States, Canada, and across Europe, including “leading retinal researchers and clinical experts, three genetic counselors, and two patient advocates” [29]. A registry coordinator (with human subject research protection certification) was also designated to act as a custodian to ensure that data entered is accurate and consistent. It should also be noted that the remainder of the registries in the review did not report on the staff required to run their registries [27, 28, 31–33, 35–38, 41].

### Consent and data access

With respect to obtaining consent prior to participating in the registry, the vast majority of identified MD patient registries did indicate that informed consent given by the patient or their caregiver (written or electronic) was required as part of the registration process (Table 3) [7, 26, 28–30, 32–37, 40]. In contrast, literature describing the Norwegian Inherited Retinal Disease Registry, the Western Australian Retinitis Pigmentosa Register, the Department of Ophthalmogenetics of the Netherlands Ophthalmic Research Institute Central Registration, the Netherlands Leber’s disease Archive, and Bradford’s Low Vision Register did not indicate if and how consent was obtained [27, 31, 38, 39, 41]. Of the registries that do describe these processes, Rosales et al. [7] reported that in addition to requiring informed consent, the NAMDC Registry also asks that participants under the legal age of majority complete an assent form. A similar procedure for participants between the ages of 12 to 17 years old was described for the My Retina Tracker Registry [29]. Furthermore, the MDCR, the AIRDR, and the My Retina Tracker Registry each expanded on their consent processes and levels of privacy afforded to their participants [28, 29, 35]. This included specifically asking participants if they consented to being contacted for participation in research, sharing their data externally or having it used for research purposes, and specific instances where their shared data would be de-identified. The My Retina Tracker Registry was the only included MD registry that had a description of how patients could revoke their consent and opt to leave the registry [29].

**Table 3:** Patient consent and stakeholder access to data within each Mitochondrial Disease (MD) patient registry.

Registry	Informed Consent	Access to Data
Australian Inherited Retinal Disease Register and DNA Bank (AIRDR) [28]	Written informed consent. Further written consent required in the case that de-identified data is required for a specific purpose	Clinicians and researchers
Bradford’s Low Vision Register [31]	NR	NR
Champ Foundation Registry (CFR) [33]	Caregivers provided electronic informed consent	External parties conducting research to develop therapies or treatments
Danish Retinitis Pigmentosa Register [36, 37]	Informed consent, unclear which format	NR
Department of Ophthalmogenetics of the Netherlands Ophthalmic Research Institute Central Registration [39]	NR	NR
European Friedreich’s Ataxia Consortium for Translational Studies (EFACTS) [32]	Written informed consent	NR
Iranian Registry for Inherited Retinal Diseases [34]	Written informed consent either provided directly from patients or their legal guardian	Clinical staff and researchers
Mitochondrial Disease Community Registry (MDCR) [35]	Unclear, but indicated that there was some form of electronic consent. Registrants were given the option of three different data sharing procedures: "allow" for data to be shared; to be "ask-"ed whenever data would be accessed; and "deny" where they indicate that their data could not be used.	Patients and researchers
My Retina Tracker Registry [29, 30, 40]	Written or electronic informed consent. For those under the age of majority, parents/guardians provided informed consent, and those between the ages of 12 to 17 years old provided assent. Registrants also chose how their data would be shared and if they were willing to be contacted by registry staff and external researchers. Consent can be changed and patients can choose to remove themselves from the registry at any time.	Clinicians, patients, and researchers
Netherlands Leber’s Disease Archive [41]	NR	NR
North American Mitochondrial Disease Consortium (NAMDC) Registry [7, 26]	Upon enrollment, written or electronic informed consent. Assent was required for registrants under the legal age of majority	Clinicians, patients, and researchers
Norwegian Inherited Retinal Disease Registry [38]	NR	Clinical staff involved in follow-up
Western Australian Retinitis Pigmentosa Register [27]	NR	Clinicians and researchers

NR, Not Reported; SLSMDS, Single Large-Scale mtDNA Deletion Syndromes.

In terms of who had access to the information once it was inputted into the registry, there were mixed findings (Table 3). Many registries indicated that researchers, clinicians, and/or clinical staff could have access to the data upon request (the NAMDC Registry, the Norwegian Inherited Retinal Disease Registry, the Iranian Registry for Inherited Retinal Diseases, the My Retina Tracker Registry, the AIRDR, the Western Australian Retinitis Pigmentosa Register, the MDCR, and CFR), but only the MDCR, the My Retina Tracker Registry, and the NAMDC Registry explicitly indicated that patients would still have access [7, 26–29, 33–35, 38, 40]. Furthermore, Fisher et al. [29] reported that after inputting their own data, the My Retina Tracker Registry participants could view graphs that show how their responses compare to the overall aggregated cohort responses. In the case of the Norwegian Inherited Retinal Disease Registry, access was restricted to only to staff that took part in clinical follow-up due to national privacy legislation [38]. Access to registry data was not described for the remaining registries in the included papers [31, 32, 36, 37, 39, 41].

### Registry outcomes

Most of the included literature outlined the impacts and outcomes that came from the establishment of these registries. Two thirds of the registries indicated that epidemiological estimates such as prevalence and incidence of disease(s) were determined based on patient data that was collected [26, 27, 30, 32, 34, 36, 38, 39]. Just under half reported that participant recruitment for various research initiatives such as clinical trials and natural history studies was made possible by engaging with patients through the registries [7, 28, 30, 32, 33]. Additionally, the NAMDC Registry was able to create a standardized diagnosis tool, the NAMDC Diagnostic Criteria [7], and the My Retina Tracker Registry was the only registry that was used to support a health economic analysis in the form of an international survey (i.e., burden of disease), as per the included literature [30]. The MDCR and the CFR were the only two registries that had reported outcomes relating to patient perspectives [33, 35]. For example, several themes emerged from the analysis of the collected data from the MDCR: registrant views on participation in research; patient priorities in relation to researchers, clinicians, and drug approval agencies; patient-identified barriers navigating the healthcare system; and impacts on overall quality of life [35]. Similarly, the CFR focused on PROs, and in addition to describing the disease progression and presentation, analysis of the surveys showed that SLSMDS severely impacts patients’ quality of life [33]. Furthermore, recommendations for healthcare team composition for optimal clinical care were made based on thematic analysis of patient responses in the CFR [33]. Only Bradford’s Low Vision Register and the Netherlands Leber’s Disease Archive did not provide any information regarding their tangible outcomes [31, 41].

### Reported registry successes, weaknesses, and suggestions

Most of the included literature reported on registry strengths or successful features, weaknesses, as well as suggestions for future registries (Table 4). Common MD patient registry strengths that were identified in the literature included being multi-center registries and recruiting from multiple avenues to avoid recruitment bias, using validated study measures, using standard terminology to increase usefulness for future international research collaborations, and acting as the first longitudinal prospective cohort of patients to characterize MDs within geographic regions [26, 27, 30, 32–34, 37, 38]. Other strong features were less commonly reported. For example, Reynolds et al. [33] reported that consultation with stakeholder groups in the creation and validation of the CFR surveys in addition to identifying their priorities took place, which was not mentioned in other included literature. Zilber and Yeske [35] highlighted that the MDCR was one of the first registries to explore patient perspectives by collecting data from this community directly, and the NAMDC’s Research Diagnostic Criteria allowed for standardization across its 17 member sites [7]. On the other hand, there were also several shared weaknesses among the identified registries. Examples include: selection and recruitment bias, resulting in overall non-generalizable findings; non-validated survey measures and unclear wording leading to participant confusion and lowering the confidence in the interpretation of results; low proportions of genetically confirmed diagnoses as well as concerns with how accurate diagnoses were; and in some cases recall bias and inconsistent levels of detail in self-reported responses from patients [7, 26, 28, 33, 36–38]. Less than half of the literature describing the included MD patient registries provided suggestions for future registries. The few that did indicated that pilot studies should be conducted prior to launching the registry as well as for novel features or projects [30, 34], consistent data checks to maintain quality standards should be undertaken [35], there should be a plan for financial sustainability for the registry [7], and direct plans of action to reduce recruitment bias (e.g., promoting remote enrollment) [26, 33]. Findings pertaining to strengths, limitations, and any recommendations were not reported for the Netherlands Leber’s Disease Archive [41], the Department of Ophthalmogenetics of the Netherlands Ophthalmic Research Institute Central Registration [39], and the Bradford’s Low Vision Register [31].

**Table 4:** Mitochondrial Disease (MD) patient registries’ reported successes, weaknesses, and suggestions.

Registry	Successes	Weaknesses	Suggestions
Australian Inherited Retinal Disease Register and DNA Bank (AIRDR) [28]	Reported genetic test results to patients (when consent was provided) for clinically relevant findings. Data is collected nationally on a yearly basis.	Since this registry was originally regional in coverage for Western Australia prior to becoming national in 2009, there is an over-representation of Western Australian participants.	NR
Bradford’s Low Vision Register [31]	NR	NR	NR
Champ Foundation Registry (CFR) [33]	As this was a multi-center registry, avoided biases that arise from single-center data collection. Consulted stakeholders for the creation and validation of surveys, and identified stakeholder priorities (patients and family; physicians and industry).	May have over-represented patients with Pearson syndrome because of a more established social media community for the former (via recruitment). The PRO measures used in the surveys were not standardized (making it hard to use this data as a “control arm in a SLSMDS clinical trial” [33 p. 303]. There was recruitment bias for registry participants; high income and well-educated families were over-represented.	To address the recruitment bias, the CFR plans “to help alleviate some of the information barriers related to diagnosis and treatment” [33 p. 307] for families that are of low SES.
Danish Retinitis Pigmentosa Register [36, 37]	Recruited from multiple avenues, increasing reach of registry within population	Patient self-reporting may be indicative of recall bias. Genetic classification was based upon clinical and pedigree collected data (e.g., issue when father is not biological father of child). One of the Danish eye clinics (most important at time) did not have diagnostic index and was therefore not used as a source of patients in registry–this limits generalizability of incidence and prevalence rates. Some potential underreporting due to the National Diagnostic Index for In-patients categorizing RP often as a secondary diagnosis as well as date limitation of Dec 31, 1987, for reporting of family members for inclusion into registry. Some evidence of selection bias (more complex patients more likely to be included). Concerns about outcome assessment due to lack of strong diagnostic tools in early 20th century. Some noted bias towards more male patients in earlier records (more likely to be seen by specialist than female patients).	NR
Department of Ophthalmogenetics of the Netherlands Ophthalmic Research Institute Central Registration [39]	NR	NR	NR
European Friedreich’s Ataxia Consortium for Translational Studies (EFACTS) [32]	Registry allowed for genetic and clinical characterization of FRDA	Inconsistent levels of detail in the self-reported responses from patients with respect to medical diagnoses and symptoms. Authors attempted to compensate by using ICD-10 to standardize diagnoses, but may “have caused additional blurring of data” [32 p. e929]	NR
Iranian Registry for Inherited Retinal Diseases [34]	Used standard terminology to increase usefulness of data and chance of global collaboration. Recruited patients using multiple methods: support groups as well through social media. To avoid selection bias, did not allow patients to self-register (e.g., internet access, literacy, aware of medical condition).	Some data from patients was self-reported; to increase confidence these responses were cross-validated against clinical records. Data was missing for ~8% of the registry data elements.	Conduct a pilot study "to identify possible barriers for implementation of the national phase in multiple cities with various geographical characteristics” [34 p. 446]
Mitochondrial Disease Community Registry (MDCR) [35]	This “is the largest study of patients with mitochondrial disease” [35 p.e16], and the first to explore patient perspectives through collection of data from patients/caregivers directly.	Survey was not validated, and all data was self-reported without any external validation. Unclear wording of survey questions led to incorrect input of answers with respect to patient and/or caregiver information, impacting interpretation of the findings. There were also inconsistencies in answers regarding diagnosis and genetic testing, and authors have indicated that future surveys will have additional data checks throughout for consistency of responses. Important to note that due to the nature of this registry, cannot calculate prevalence of any primary mitochondrial disease as diagnosis was not required, nor can confirm that all participants truly had some form of a mitochondrial disease. With respect to generalizability, majority of participants were White, female, and living in the United States, and therefore findings were not generalizable.	It is possible to get the patient perspectives outside of traditional qualitative research that may not be within the capacity of patient organizations and associations by asking open-text field questions. Additionally, it was strongly recommended to implement consistent data checks in future registries to maintain quality standards.
My Retina Tracker Registry [29, 30, 40]	Used validated PROs measures and tools. Participants in the United States were eligible for a pilot program for the inclusion of genetic counsellors who were able to communicate test results to patients and maintain trust and transparency. One genetic testing provider was used to ensure data consistency and reduce administrative workloads.	The pilot program had associated challenges, such as non-registrants being included, clinicians not consulting with most recently collected data in the registry, requiring ethics approvals in academic research centers prior to upload of data into the registry. These resulted in delays for patients to receive their results.	The pilot program ended up being removed as direct aspect of the registry and became its own entity separate from the registry to reduce administrative stress on the registry.
Netherlands Leber’s Disease Archive [41]	NR	NR	NR
North American Mitochondrial Disease Consortium (NAMDC) Registry [7, 26]	Registry allowed for genetic and clinical characterization of MDs in North America. Established diagnostic standard, NAMDC Research Diagnostic Criteria	Suspected recruitment bias as only participants who lived close to NAMDC partner centers enrolled, excluding those who live in remote areas. Also noted that there was a racial disparity in the composition of the participants; there was a higher than expected proportion of White patients but authors could not verify if this was due to recruitment bias. Ascertainment bias was also highlighted due to a higher-than-expected proportion of participants diagnosed with MELAS as some of the NAMDC sites conduct research on this disorder.	Ensure that there is a plan for financial sustainability (e.g., support from partner organizations and industry) and promote remote enrollment
Norwegian Inherited Retinal Disease Registry [38]	Was the first registry in Norway to provide data to determine prevalence of IRDs in this population, alongside genetic and clinical characterizations.	A low proportion of registrants had genetically confirmed diagnoses, the age distribution skewed to a younger population, and the geographic coverage was incomplete.	NR
Western Australian Retinitis Pigmentosa Register [27]	Consistent and systematic collection of data, use of accurate measures, rigorous quality control for data collection, support from associations and clinicians, the creation of a prospective longitudinal study allowed for better understanding of disease progression and trends, strong effort to establish genetic status of registrants.	NR	NR

FRDA, Friedreich’s Ataxia; ICD, International Classification of Diseases; IRD, Inherited Retinal Disease; MELAS, Mitochondrial Encephalopathy, Lactic Acidosis, and Stroke-like episodes syndrome; NR, Not Reported; PRO, Patient Reported Outcome; SES, Socioeconomic Status; SLSMDS, Single Large-Scale mtDNA Deletion Syndromes.

### Stakeholder consultations

Several stakeholders were consulted for this scoping review between October 2020 and January 2021. The purpose of these consultations was to gain a deeper understanding of MD patient, caregiver, and community perspectives (especially in Canada), to learn from people who have previous experience with patient registries, to hear what an ideal registry would encompass, as well to understand how best to serve the Canadian MD community. These meetings included conversations with patient caregivers, advocates, researchers with previous patient registry experience, MITO2i, and MitoCanada. From these conversations, two major themes emerged.

### Theme 1: Barriers to participation

#### Lack of communication

Often, a barrier for patients to participate or consider joining a registry is the lack of communication, follow-up, and ultimately respect that is not afforded to the time and effort they give. A common message that came across from either our stakeholders’ personal experiences or from what they know from the MD community is that when patients get cut out of the research process, they often wish that they had not consented to sharing their data or time in the first place. This wall of silence from the researchers’ side is extremely frustrating and disheartening. To combat this unfortunate norm and build trust with the patient community, it was strongly suggested that patients must be considered equal and active partners within any subsequent research that takes place through it. Ongoing, two-way communication and the intentional dissemination of findings is key.

#### Patient data ownership

Another related barrier was surrounding the topic of data ownership. It was highlighted that it is important for researchers to recognize that the knowledge (e.g., the data set, the samples, etc.) isn’t owned by them, but rather it is owned by patients, their families, and caregivers. Moreover, patients want to know if and how their data is being used, what the results are, what the potential research impact will be, in addition to when new therapies become available. Patients want to help in advancing the current body of knowledge and work with researchers. Once again, respecting the role of patients within the research process and maintaining a channel of communication was put forward by several stakeholders.

#### Registry modality

While most discussions surrounding MD patient registries assumed access to a computer for the end user, it was noted that modality is not something to take for granted. One of the patient advocates indicated that it is important to have more than one method; surveys that are available electronically should also be shared on paper or by phone interviews. It is essential to offer a method that is most accessible and convenient to the patient so as to avoid systematic exclusion (i.e., recruitment bias).

### Theme 2: Registry planning

#### Registry objectives

The goals of the registry must be made explicitly clear, and they should be established through engagement of all relevant stakeholders (e.g., patients, clinicians, and researchers). In this way, research priorities can align with patient priorities. These goals will dictate the structure and design of the registry and identify who will be entering data, what kind of information will be captured, and which standardized measures will be appropriate. For example, while clinicians may be able to enter more accurate data regarding a patient’s medical condition, only the patient can truly describe their quality of life and lived experiences. There is value in these different types of data, however, it is important to ensure that only the data relevant to registry objectives are captured so as not to take up unnecessary time from the registrants. Additionally, it is important to take the time to ensure that the platform that is chosen to host the registry meets all the requirements outlined by the registry goals, as once one is chosen, there is often no going back.

#### Data standards

Several stakeholders raised the issue that, when possible, it is beneficial to use validated data standards. It is important to take the time to understand which measures are accepted by researchers, industry partners, government regulatory bodies, as well as internationally. Moreover, the choice of standards will once again depend on the overall goal of the registries and what kind of information is trying to be captured.

#### Funding

The cost of running an MD registry came up in a few of the consultations. Stakeholders indicated that it is important to identify who are the potential sources of funding for the registry (e.g., the pharmaceutical industry, government) and to integrate this into the long-term planning of the registry.

#### Data quality

It was suggested that prior to opening a registry to patients, a pilot study should be run. This would ensure that the registry interface is user-friendly, and that the questionnaires are clearly worded, understandable to the user, and of appropriate length. Furthermore, after officially opening the registry up to users, constant evaluation of the data captured is vital as a part of quality control and to be able to address any problems that arise in a timely manner. In these ways, one can have a higher chance of a successful registry and useable high-quality data.

#### Privacy, security, and governance

In almost all the stakeholder consultations, privacy regulations, security, and data governance were brought up, as personal health data is collected in patient registries. In addition to navigating the various institutional, provincial/territorial, and national regulations within Canada to ensure patient privacy is maintained, this can become even more complicated should the registry be open to international participants. However, there is much value to be gained with a wider cohort of registrants through the facilitation of international collaborations. As such, it was strongly suggested that clear policies surrounding data governance must be set in place as well as ensuring that the registry complies with international legislation surrounding data privacy and security.

## Discussion

To our knowledge, this scoping review is the first to provide an overview of existing MD registries worldwide. Many MDs are rare diseases. Therefore, the limited number of patients and the disease-specific natural history data, the complexity of phenotypes, and inconsistent responses to treatments between MD patients call for aggregated data sources such as a comprehensive patient registry [43]. A good quality registry is an invaluable tool for gathering, storing, and managing patient data [13]. Aligning with what we found in the registries described by the literature included in our scoping review, Williams and Karpelowsky’s [13] review of medical registries reported that the collected data in the registries can be used for various purposes such as etiological, epidemiological, and health outcomes research. This data can also be used to assess the efficacy of new treatments for a specific patient population [13]. Hence, registries provide researchers, practitioners, and policymakers with the required information to improve the quality of care and treatment. For patients, caregivers, and patient-focused organizations, a registry can also be a knowledge translation source for understanding the natural history and progression of a disease, allow them to be involved in the creation of best practice and treatment guidelines, as well as provide them with timely research updates and notices of novel treatments and therapies [11, 44]. Our findings revealed that despite the proven benefits of disease registries in facilitating research, making targeted health policies, and improving the quality of life of patients, there is a limited number of MD registries around the globe. However, considering the publication date of the included studies in this review, it seems that the necessity of these registries has been more recognized recently as 13 of the 17 included studies were published in the last decade. As it was one of the objectives of this scoping review, we paid special attention to the involvement of the Canadian MD patients. Although there are some established patient contact registries that provide information to patients and facilitate recruitment for research (e.g., clinical trials) [14, 15], there is no formal clinical MD patient registry in Canada. However, there were three registries based in the United States with international coverage that included some Canadian patients [7, 30, 35]. Lack of an aggregated data source of the Canadian MD patients was one of the main concerns of the patient advocates and caregivers that we consulted. Hence, our findings highlight the necessity of the development of the Canadian MD registry to serve the Canadian population more comprehensively. As such, we have provided recommendations for the creation of such a registry based on our findings from the included literature as well as our stakeholder consultation.

### Establishing clear registry objectives

Defining clear objectives and goals early in the process of the development of a patient registry is key to its success and longevity [12]. The objectives should be precise, specific, and achievable. The objectives of a registry define which patients should be included, what geographical area should be covered, and what type of information should be collected [12]. As described by Gliklich et al. [11], rare disease registries can have wide variety of objectives such as to connect affected patients, caregivers, and health care practitioners, to understand the natural history of particular diseases, as well as to support different types of research and treatment development. Accordingly, almost all of the included literature in this scoping review described registry objectives that fell throughout the spectrum described above, from characterizing MDs through patient perspectives in the MDCR [35], to supporting the conduct of translational research on MDs in the NAMDC Registry [7, 26]. The importance of determining the explicit goals was one of the sub-themes that emerged from our stakeholder’s consultation (i.e., *registry objectives*). More specifically, stakeholders expressed that the goals of a registry should be defined collaboratively with involvement of patients, clinicians, researchers, and funding bodies. Hence, clear, robust, and achievable objectives that are developed through the engagement of different stakeholders are a significant factor that contributes to the success of a registry and should be considered as one of the first steps in the development of a Canadian MD patient registry.

### Sustainable source of financial and intellectual support

One reason for the limited number of MD registries worldwide can be the multi-disciplinary nature of the development of such a registry. Generally, establishing a patient registry requires the involvement of different stakeholders and sufficient funding [12]. These requirements were evident in the included registries in this scoping review as most of them were associated with multiple academic or clinical institutes, patient advocacy foundations, and government organizations to obtain the required intellectual or financial support. For example, Rosales et al. [7] described that the steering committee of the NAMDC Registry is establishing strategic plans to collaborate with various non-for-profit and industrial organizations to ensure long-term sustainable funding and infrastructures. The importance of the identification of sustainable sources of *funding* was also one of the sub-themes that emerged from our stakeholder consultation, and it was recommended that it should be one of the first main steps in the development of the Canadian MD patient registry. As such, collaboration with various organizations to ensure viable supports that guarantee the longevity of the registry should be one of the main factors in the establishment of any future Canadian MD patient registry.

### Diagnostic odyssey and engaging in two-way communication

Another reason for the scarcity of MD registries, that is more specific to this particular group of diseases, can be the difficulty in recruitment of MD patients and their caregivers. This can be due to the difficulty in diagnosis of MDs [8]. Depending on the involved genes, MDs can have a wide variety of presentations that cause a patient to experience years of consultations with several different physicians to receive an appropriate diagnosis [8]. Moreover, this diagnostic odyssey paired with limited available and effective treatments may leave MD patients and their caregivers tired and reluctant to take any active steps to participate in a registry. Furthermore, another hurdle in recruiting MD patients in a registry that emerged from our stakeholder consultation was patients’ and their caregivers’ unsatisfied expectations from participation in such a registry. Patients and caregivers have a fundamental role in the success of a registry. When patients participate in a registry or a particular research project, they place a lot of hope and trust into this involvement. MD patients’ caregivers involved in our stakeholder consultation were expecting ongoing two-way communication by consenting to participate in a registry. They were disappointed when the investigators stopped communicating with them (i.e., sub-theme *lack of communication*) after they shared their personal and clinical information. Patients and caregivers will be more willing to participate in a registry if they find themselves as true partners in the registry and are informed of the impacts of their participation on research, clinical outcomes, or policies. Among studies included in this scoping review, Fisher et al. [29] emphasized the importance of communication initiatives in promoting patients’ participation and ongoing cooperation with the registries. Also, in the description of the MDCR, which was a registry based on patients’ perspectives, Zilber and Yeske [35] accentuated that the doctor-patient relationship has a significant impact on MD patients’ willingness to participate in the registries. Hence, ongoing two-way communication with patients and their caregivers is a key to the success of a registry and should be considered when establishing a Canadian MD patient registry.

### Consider multiple recruitment methods to avoid bias

A challenge that was mentioned in a few registries was the observation of recruitment bias. Specifically, the CFR shared that their patient population was mostly of high socioeconomic status (SES, high income and highly educated) [33], and the NAMDC Registry shared that they suspected that they were missing patients who did not live near their recruitment centres [26]. To tackle these challenges, the CFR will be sharing information regarding diagnosis and treatment to bridge what they suspect to be a knowledge gap for lower SES families that hinders their enrollment [33], and the NAMDC Registry has been promoting remote recruitment to capture their missing patient populations [26]. The Danish Retinitis Pigmentosa Register [37] anticipated that this may be an issue and tackled this bias by recruiting patients from multiple avenues (e.g., national patient organizations, hospitals, private clinics, and physicians), which increased the reach of their registry. These actions fall in line with what our stakeholders have shared with respect to the importance of the sub-themes of (the *lack of*) *communication* with patients as well as considering more than one *modality* (in this case for recruitment) for the registry to avoid bias. These different strategies all speak to the importance of maximal recruitment to ensure that the registry has as much data as possible to be useful to the different stakeholders (e.g., patients, researchers, clinicians) [13], and should be considered in the development of a Canadian MD patient registry.

### Development of a consensus-based diagnostic criteria

Data collected from clinical registries are crucial in establishing diagnostic guidelines and management plans for diseases, particularly with MDs which often present with multiple clinical symptoms. As previously mentioned, participants in some registries can be enrolled based solely on clinical manifestations, and genetic testing is not always required. For example, patients in the NAMDC Registry had to be diagnosed by an experienced clinician at a participating site in order to become eligible for inclusion in the registry [26]. However, the patient can submit a biosample for genetic analysis at the time of consent, although this is not mandatory [7]. It was also acknowledged that the diagnostic process is extremely complex and that many participants in the NAMDC Registry do not fit into the classical mitochondrial phenotypes, “with multisystemic syndrome [being] the most common diagnosis” [26]. Since MDs have such a wide range of symptoms, it was recommended by Rosales et al. [7] that common and agreed-upon criteria be used for the diagnosis and classification of patients, such as the NAMDC Research Diagnostic Criteria that they developed. A survey of Canadian physicians who treat MD patients also identified that there was a need to continue developing and improving diagnosis and diagnostic care [45]. As such, it will be important to engage with clinicians and physicians when considering available diagnostic criteria or if opting to pursue the creation of consensus-based diagnostic criteria through a future Canadian MD patient registry.

### Adoption of international data standards

It has been suggested that in order for a registry to operate successfully, it must have the following characteristics: “consistency in record keeping…and [rigorous] technical quality control so that serial data may be compared accurately over time” [27]. Experts in the field have also stressed the importance of harmonizing global mitochondrial patient registries and data sharing for clinical research [43]. It was also clear from our scoping review that different registries are using various standards that might impact interoperability and data sharing down the line. In addition to being more time consuming and costly, trying to “[aggregate] datasets from different sources [in non-standardized formats will] compromise data quality and integrity and lead to interpretation errors that would limit [data] accuracy, accessibility, and reusability” [43]. It was also mentioned in our stakeholder consultation that the use of validated *data standards* is fundamental especially if different organizations use different data systems. Through the implementation of a unified, widely accepted system for structured data collection and sharing, research can be facilitated and accelerated, maximally benefiting all stakeholders (e.g., patients, researchers, and clinicians) [43]. In connection with this, there has been an effort towards developing “FAIR” (findable, accessible, interoperable, reusable) data standards to make data more shareable as well as building a more transparent data-sharing model for the community [43]. Utilizing disease-specific Global Unique Identifiers (GUIDs) and their derivatives for secure data integration is one example of applying FAIR data standards. In our review, the My Retina Tracker Registry is the only registry that integrates GUIDs. In this way the data on each study participant can be exchanged without exposing personal identifiable information, and the data on each patient can be linked between independent databases that conform to the GUID standard [29]. Applying international data standards that follow the principles of FAIR should be considered when developing a Canadian MD patient registry to support the ongoing initiative of global MD patient registry harmonization and facilitate potential future international research collaborations.

### Conducting pilot studies to improve data quality and collection

In addition to using data standards, it is equally important to ensure that the data that is collected is of high quality. Primary data sources include direct engagement with patients, caregivers, or clinicians to solicit information, while secondary data sources are data that were previously collected for other purposes but have value for the patient registry (such as insurance claims, e-health records) [11]. For the former, data collection will often be through a survey, whether electronically, by telephone, or by paper [11]. Surveys may include previously validated questionnaires and tools. It has been highlighted in literature describing rare disease registries [11, 13, 35, 46], as well as by our stakeholders (*data quality* sub-theme), that pilot studies should be conducted to increase the chances that high quality data is collected. A pilot study can ensure that the surveys used for data collection are clear, user-friendly and understandable, are not extremely time-consuming and burdensome to patients, caregivers or clinicians, and that the information that is being requested is reasonably accessible to the respondent [11, 35]. Of the included registries in this scoping review, it was reported that the MDCR ran into issues with the quality of their data because it was unclear if their survey questions were targeting patients or their caregivers, resulting in difficulty with data interpretation and usability [35]. Encouragingly, the Iranian Registry for Inherited Retinal Diseases [34] is first being run as a pilot study in only two cities to try and capture such issues as it is the first registry for IRDs in Iran. For a Canadian context, it will be important to also run such pilot studies, to ensure that primary data collection surveys methods are appropriate and functional to avoid unexpected pitfalls in the long-term. Working in collaboration with the already established Patient Contact Registry from MitoCanada and Lumiio as well as LHON Canada’s contact registry may be beneficial to understand what has worked thus far for the data they have collected within a Canadian population when developing a Canadian MD patient registry [14, 15].

### Using different modalities for primary data collection

Implementing multiple modalities for primary data collection has been highlighted as an important consideration for patient registries and may take many forms, such as electronic or paper-based surveys as well as interviews conducted in-person or by telephone [11]. This has been echoed in our stakeholder consultation, and more specifically it was suggested that the *modality* used for data collection must be the preferred and most accessible method for the patient, centering them and their needs within the research process. As previously identified, most registries indicated that healthcare staff, clinicians, or researchers collected patient data through interviews or by consulting patient health records and input the data themselves into the registry, with exception of the My Retina Tracker Registry, the MDCR, and the CFR that indicated that patients or their caregivers were able to provide and enter information themselves [29, 33, 35]. However, most of the included literature did not indicate that there was a choice of modality when it came to data collection other than the My Retina Tracker Registry where there was the option of completing data collection by an electronic or paper-based survey. In contrast, the Iranian Registry for Inherited Retinal Diseases specified that they did not allow patients to input data themselves to avoid what was perceived to be selection bias due to the lower levels of literacy, access to the internet, and patients who knew their diagnoses within the two Iranian cities that were initially included in the registry [34]. Instead, the registry’s steering committee decided that trained retina specialists would be better suited for data entry after interviewing patients [34]. While the registry designers were tailoring their recruitment and data entry strategies to their patient community to try and be as inclusive as possible, this may have inadvertently disempowered patients within the process as it removes their ability to choose how to engage within the registry or provide them with a modality that is most accessible and preferred to them. However, Sabbaghi et al. [34] did not expand on this patient perspective within the Iranian Registry for Inherited Retinal Diseases so this remains unclear. For a Canadian MD patient registry, it will be important to engage with patients to understand what their capacities are for participating in primary data collection and provide them multiple modalities to best meet patient preferences and support accessibility.

### Evaluating data consistently upon opening the registry

As a complement to pilot testing, consistent evaluation of the quality of registry data is vital once the registry is fully operational (i.e., open to registrants). Kodra and colleagues [12] specifically recommend that data should be monitored at consistent intervals (e.g., for accuracy, instances of duplication, completeness of data, irregularities) and that this should be documented in the form of reports. This was echoed in Gliklich et al.’s [11] user guide for patient registries, as well as during our stakeholder consultation regarding *data quality* such that issues that arise can be dealt with in a timely manner. In fact, this was a recommendation from the experiences of the MDCR. More specifically Zilber and Yeske [35] indicated that data quality checks would be implemented in future endeavors to ensure that patient responses within the registry’s survey were both complete and consistent. Data evaluation will be vital and should be clearly outlined as part of the operational planning in a future Canadian MD patient registry.

### Supporting patient autonomy and data ownership through informed consent

Informed consent has been highlighted as a vital part of the registry recruitment process, providing an additional layer of safety for patients as well as supporting patient autonomy [10, 12, 47]. More specifically, Kodra and colleagues [12] indicate that effective informed consent should provide the patient with information on the objectives of the registry, what data they will be asked to provide and how the registry will be using and storing it, who will have access to their data (including any third parties), how the patient can get access to their own data, and how the patient can revoke their consent should they choose. Within the identified MD patient registries in this scoping review, the few that described these individual items beyond obtaining informed consent were the My Retina Tracker Registry, the MDCR, and the AIRDR [28, 29, 35]. These registries required further consent when data would be used by a third party or would be de-identified, giving more agency to the patients as well as keeping the research process transparent. In terms of revoking consent, of the included articles only Fisher et al.’s [29] paper described how patients could remove themselves from the My Retina Tracker Registry. These practices fall in line with identified sub-themes from the stakeholder consultation, including combatting the *lack of communication* and supporting *patient data ownership*. It is critical to support the roles of patients within the research process and regard them as equal collaborators by allowing them to choose if and how involved they would like to be, recognize that the data they share is owned by them, as well as informing them of how their data will be used to advance the field. The steps taken by the three aforementioned registries support this call for transparent patient engagement in different aspects of MD registries. Patient autonomy and data ownership will be important considerations for a future Canadian MD patient registry.

### Complying with privacy legislations

When creating a registry, it is important to ensure that privacy regulations and legislation are being followed. With the exception of the My Retina Tracker Registry [30], Norwegian Inherited Retinal Disease Registry [38], and the Danish Retinitis Pigmentosa Register [36], this was not discussed in the literature describing the remaining registries within this scoping review. In the case of a future Canadian MD patient registry that may hold the genetic information of patients, it will be paramount to securely house this captured data and not release it to any third parties (without explicit consent) to protect these patients from discriminatory practices as outlined in the *Genetic Non-Discrimination Act* [48]. An additional suggestion that was brought up during the stakeholder consultations was considering international privacy legislation should a registry be international in coverage (or wish to have the option for future international expansion or collaborations). This has been echoed within patient registry literature, with emphasis that registry policies on data governance and privacy should fall in line with international laws when possible [10, 12]. An example of legislation to keep in mind should a Canadian MD patient registry be founded that wishes to expand internationally is the *General Data Protection Regulation* [49], which covers data governance as well as informed consent across the European Union [12]. Incidentally, this legislation is what the current Canadian federal privacy legislation, the *Personal Information Protection and Electronic Documents Act*, is being reviewed against to ensure that data can move between Canada and the European Union [50].

### Limitations

This scoping review has some limitations. First, only literature that was published in English was included in this review due to limited access to translation resources. Additionally, we included literature published in only two academic databases (PubMed, CINAHL). This may have inadvertently excluded MD patient registries and may have been a contributing factor as to why registries were mainly identified in North America, Europe, Australia, and West Asia. Future research should consider the inclusion of literature published in other languages as well as expanding the search into additional academic databases. Second, only articles published in journals and registry websites could be included in this scoping review, while the official websites of registries were not examined as sources of data. This may have resulted in extracted information from the included literature that was not up to date, as well possibly missing information that is available on a registry’s website. Furthermore, we may have inadvertently excluded MD registries that do not currently have published articles in English by not including registry websites as a source of information. Moreover, our recommendations are based on a limited number of studies in addition to our stakeholder consultation. Next steps in this research include fully analyzing registry websites systematically to identify any missed MD registries, and gain a fuller understanding of what the designs, strengths, and limitations of the current MD patient registries worldwide are. Additionally, we acknowledge that there may be details pertaining to MD patient registries that were not discussed in detail within the included literature or may not have been reported within the registries’ websites. As such, we suggest that future research involves engaging with all existing registries directly through quantitative surveys (with open-ended questions), semi-structured interviews or focus groups, or an all-encompassing mixed-methods exploratory study. Finally, while our stakeholder consultation included MD patient caregivers, advocates, and researchers, we, unfortunately, were not able to meet with clinicians, who may have shared different expectations and experiences in relation to MD patient registries. Our findings from our stakeholder consultation would have been strengthened by engaging with this group of stakeholders as we would have been able to examine commonalities and divergences more fully between patients, researchers, and clinicians who each have their own biases, perspectives, and vested interests within the MD community in relation to what a Canadian MD registry could take shape as. Our scoping review is a first step at capturing some of these different perspectives, and future research should include clinicians in addition to patients and researchers to catalogue the breadth of views and be as inclusive as possible.

## Conclusion

MDs are a complex group of diseases for which patient registries have proven to be useful tools. Registries can greatly facilitate many types of research, connect and engage stakeholders within a shared community (i.e., patients and caregivers with researchers and clinicians), improve clinical practice, and support treatment development. The creation of a national MD patient registry in Canada would be beneficial to this community. This scoping review is one of the first steps in supporting this initiative, in addition to filling a knowledge gap as there is yet to be any review or narrative synthesis describing MD patient registries. Through this review, we identified numerous MD patient registries around the world, the majority of which were established in North America, Europe, Australia, and West Asia. This scoping review was also able to identify information pertaining to these registries’ designs, the types of data they are collecting, who the end users of the registries are, as well as what their outputs and impacts were. Through the examination of this literature as well as the consultation of stakeholders (patient carers, advocates, and MD researchers), we were able to provide recommendations for registry planning, including clinical, technical, and ethical considerations, suggestions for patient engagement and recruitment, as well as potential challenges to overcome. These recommendations in turn should be used to support the Canadian MD community in the creation of a national MD registry.

## Supporting information

S1 Table Preferred Reporting Items for Systematic Reviews and Meta-Analyses Extension for Scoping Reviews (PRISMA-ScR) checklist. (DOCX)

S2 Table Search strategy for databases used in this scoping review. (DOCX)

S3 Table List of included primary mitochondrial diseases. (DOCX)

S4 Table Items that were extracted during data mapping from the included articles. (DOCX)
