# Supplementary material for: Mitochondrial disease registries worldwide: A scoping review
Source: PLoS One. 2022 Oct 27;17(10):e0276883. doi: 10.1371/journal.pone.0276883 (PMC9612561; doi:10.1371/journal.pone.0276883)
Supplement: S2 Table — (DOCX) [file pone.0276883.s002.docx]

**S2 Table. Search Strategy for Databases Used in this Scoping Review.**

| **Database** | **Search strategy** | **Initial search (date)** | **Updated search (date)** |
| --- | --- | --- | --- |
| PubMed | ("Mitochondrial Diseases"[Mesh] OR "Mitochondrial Myopathies"[Mesh] OR "Mitochondrial Encephalomyopathies"[Mesh] OR "Mitochondria"[Mesh] OR "Barth Syndrome"[Mesh] OR "Visceral myopathy familial external ophthalmoplegia" [Supplementary Concept] OR "mitochondrial genetic disorders"[tw] OR "mitochondrial DNA deletion"[tw] OR "mitochondrial DNA duplication"[tw] OR "mitochondrial disease*"[tw] OR "mitochondrial depletion"[tw] OR "mitochondrial respiratory chain complexes"[tw] OR "mitochondrial disorder*" OR "mito"[tw] OR "KSS"[tw] OR "LHON"[tw] OR "MELAS"[tw] OR "MIDD"[tw] OR "MDDS"[tw] OR "MERRF"[tw] OR "MNGIE"[tw] OR "NARP"[tw] OR "CPEO"[tw] OR "ophthalmoplegia"[tw] OR "Mitochondrial myopath*"[tw] OR "Oxidative Phosphorylation Deficienc*"[tw] OR "Respiratory Chain Deficienc*"[tw] OR "Electron Transport Chain Deficienc*"[tw] OR "mitochondrial encephalomyopath*" OR "mitochondrial encephalopath*"[tw] OR "cytochrome‐c oxidase deficiency"[tw] OR "Ubiquinol‐Cytochrome‐c reductase"[tw] OR "retinitis pigmentosa"[tw] OR "pyruvate dehydrogenase complex deficiency disease"[tw]) AND ("Registries"[Mesh] OR "Databases, Genetic"[Mesh] OR "Information Systems"[Mesh] OR "Medical Records"[Mesh] OR "Data Collection"[Mesh] OR "Records"[Mesh] OR "patient registr*"[tw] OR "clinical registr*"[tw] OR "clinical data registr*"[tw] OR "disease registr*"[tw] OR "outcomes registr*"[tw] OR "patient database"[tw] OR "patient cohort"[tw] OR "medical registr*"[tw] OR "e-health record"[tw] OR "diseases registr*"[tw] OR "registr*"[tw]) | 3590 records identified (March 24, 2021) | An additional 162 records identified (January 26, 2022) |
| CINAHL | ((MH “Mitochondrial Diseases+”) OR (MH “Ophthalmoplegia, Chronic Progressive External+”) OR (MH “Mitochondrial Encephalomyopathies+”) OR (MH “Mitochondrial Myopathies+”) OR “mitochondrial disease” OR (MH “Leigh Disease”) OR “leigh disease” OR “Leber hereditary optic neuropathy” (MH “Mitochondria+”) OR (MM “Kearns-Sayre Syndrome”) OR (MM “Mitochondrial Neurogastrointestinal Encephalopathy Syndrome”) ) AND (  (MH “Registries, Disease”) OR (MH “Clinical Trial Registry”) OR “registry” OR (MH “National Program of Cancer Registries”) OR (MH “Electronic Health Records+”) OR (MM “Clinical Data Repository”) OR (MM “Public Reporting of Healthcare Data”) OR (MM “Data Collection, Computer Assisted”) OR (MH “Data Warehouse”) OR (MH “Data Collection+”) OR (MH “Data Analysis+”) OR (MH “Data Management+”) OR (MH “Data Mining”) OR “clinical data” OR (MH “Prospective Studies+”) OR (MM “Concurrent Prospective Studies”) OR (MM “Nonconcurrent Prospective Studies”) OR “diseases registr*” OR “e-health record” OR “medical registr*” OR “patient cohort” OR “patient database” OR “outcomes registr*” OR “disease registr*” OR “clinical data registr*” OR “clinical registr*” OR “patient registr*”) | 309 records identified (March 24, 2021) | An additional 14 records identified (January 26, 2022) |
