# Supplementary material for: Mitochondrial disease registries worldwide: A scoping review
Source: PLoS One. 2022 Oct 27;17(10):e0276883. doi: 10.1371/journal.pone.0276883 (PMC9612561; doi:10.1371/journal.pone.0276883)
Supplement: S3 Table — (DOCX) [file pone.0276883.s003.docx]

**S3 Table. List of Included Primary Mitochondrial Diseases.**

| **Category** | **Terms** |
| --- | --- |
| Broad terms relating to mitochondrial diseases | - Mitochondrial Diseases - Mitochondrial Myopathies - Encephalomyopathies - Oxidative Phosphorylation Deficiencies - Respiratory Chain Deficiencies - Electron Transport Chain Deficiencies |
| Specific Primary Mitochondrial Diseases^a^ | - Barth Syndrome - Visceral Myopathy Familial External Ophthalmoplegia - Friedreich’s Ataxia (FRDA or FA) - Leber Hereditary Optic Neuropathy (LHON) - Mitochondrial Encephalopathy, Lactic Acidosis, and Stroke-like episodes (MELAS) - Myoclonic Epilepsy with Ragged Red Fibers/Myoencephalopathy Ragged-Red Fiber Disease (MERRF) - Fukuhara Syndrome - Maternally Inherited Diabetes and Deafness (MIDD) - Mitochondrial DNA Depletion Syndrome (MDDS) - Alper’s Syndrome - Mitochondrial Neurogastrointestinal Encephalopathy Syndrome (MNGIE) - Neuropathy, Ataxia and Retinitis Pigmentosa (NARP) - Retinitis Pigmentosa (RP) - Leigh Disease/Syndrome - Chronic Progressive External Ophthalmoplegia (CPEO) - Kearns-Sayre Syndrome (KSS) - Pearson’s Syndrome - Dominant Optic Atrophy (DOA) - Hereditary Sensory Paraplegia (HSP) - Alpers-Huttenlocher Syndrome - Progressive External Ophthalmoplegia (PEO) - Sensory Ataxia Neuropathy, Dysarthria, Ophthalmoplegia (SANDO) - Pyruvate Dehydrogenase Complex Deficiency Disease - Pyruvate Carboxylase Deficiency Disease |

^a^While Single Large-Scale mtDNA Deletion Syndromes (SLSMDS) is not found within this table, it was included in this review as it is a group of diseases that includes KSS, CPEO, PEO and Pearson’s Syndrome [1].

# References

1. Broomfield A, Sweeney MG, Woodward CE, Fratter C, Morris AM, Leonard JV, et al. Paediatric single mitochondrial DNA deletion disorders: an overlapping spectrum of disease. J Inherit Metab Dis. 2015;38(3):445–57.
