# Supplementary material for: Mitochondrial disease registries worldwide: A scoping review
Source: PLoS One. 2022 Oct 27;17(10):e0276883. doi: 10.1371/journal.pone.0276883 (PMC9612561; doi:10.1371/journal.pone.0276883)
Supplement: S4 Table — (DOCX) [file pone.0276883.s004.docx]

**S4 Table.** **Items that were Extracted During Data Mapping from the Included Articles.**

| **Category** | **Items Extracted** |
| --- | --- |
| Bibliographic information and article characteristics | - DSR generated reference number for article - First author and year of publication - Source of funding for the article/paper |
| Registry characteristics | - Name of registry - URL of official registry website - Type of registry - Year of establishment - Is the registry still active? (yes/no)   - If no, until what year was it active? - Funding source(s) - Associated biorepository? (yes/no) - Country/ies of conduct - Recruitment source - Purpose and/or objectives of the registry - Coverage (regional/national/international)   - If international coverage, included countries - Included MDs - Any non-MDs included? (yes/no)   - If yes, which ones? (general category of disease) - Which participants are included? (e.g., patients, parents of patients, caregivers, etc.) - Number of participants   - Are any Canadian? (yes/no) If yes, how many?   - Are there any participants registered in other registries? |
| Technical details | - Platform used - Staffing required to run the registry - Are standardized measures used? (yes/no)   - If yes, which ones? - How was information collected? (survey/clinical visit/research project/interview etc.) - Who was involved in data collection? (clinicians/researchers/patients/caregivers etc.) |
| Clinical aspects | - Was a diagnosis required? (yes/no)   - If yes, how was it confirmed?   - Were suspected cases allowed? - Type of information collected (pedigree/genetic/clinical/PROs etc.) |
| Ethics, privacy, and impacts | - How was informed consent obtained? - Who has access to registry data? |
| Impacts, successes, challenges, suggestions | - What are the registry impacts (i.e., outcomes)? (e.g., recruitment for clinical trials, epidemiological studies, health policy changes etc.) - What are the reported registry strengths? - What are the reported registry weaknesses? - What are the reported recommendations? |
| Optional | - Additional comments from review authors (e.g., reviewer-identified strengths and weaknesses of registry) |

DSR, Distiller Systematic Review; MD, Mitochondrial Disease; PRO, Patient Reported Outcome.
